# Supplementary material for: Resolving the full spectrum of human genome variation using Linked-Reads
Source: Genome Res. 2019 Apr;29(4):635–45. doi: 10.1101/gr.234443.118 (PMC6442396; doi:10.1101/gr.234443.118)
Supplement: Supplemental Material [file supp_gr.234443.118_Supplemental_longranger-2.2.zip › longranger-2.2.tar.bz2!/lr2.2-public/lib/stan/cmdstan/stan/doc/api/html/rev_2functions_2fabs_8hpp_source.html]

Stan: src/stan/agrad/rev/functions/fabs.hpp Source File


|  |  |
| --- | --- |
|  | Stan  2.5.0  probability, sampling & optimization |


- Main Page
- Related Pages
- Namespaces
- Classes
- Files

- File List
- File Members

All Classes Namespaces Files Functions Variables Typedefs Enumerations Enumerator Friends Macros Pages

- src
- stan
- agrad
- rev
- functions

fabs.hpp

Go to the documentation of this file.

1 #ifndef STAN\_\_AGRAD\_\_REV\_\_FUNCTIONS\_\_FABS\_HPP

2 #define STAN\_\_AGRAD\_\_REV\_\_FUNCTIONS\_\_FABS\_HPP

3

4 #include <stan/agrad/rev/var.hpp>

5 #include <stan/agrad/rev/vari.hpp>

6 #include <stan/agrad/rev/internal/precomp\_v\_vari.hpp>

7 #include <stan/agrad/rev/operators/operator\_unary\_negative.hpp>

8 #include <stan/math/constants.hpp>

9

10

11 namespace stan {

12  namespace agrad {

13

53  inline var fabs(const var& a) {

54  using stan::math::NOT\_A\_NUMBER;

55  // cut-and-paste from abs()

56  if (a.val() > 0.0)

57  return a;

58  else if (a.val() < 0.0)

59  return var(new neg\_vari(a.vi\_));

60  else if (a.val() == 0)

61  return var(new vari(0));

62  else

63  return var(new precomp\_v\_vari(NOT\_A\_NUMBER,a.vi\_,NOT\_A\_NUMBER));

64  }

65

66  }

67 }

68 #endif

stan::agrad::fabs

fvar< T > fabs(const fvar< T > &x)

**Definition:** fabs.hpp:18

precomp\_v\_vari.hpp

vari.hpp

stan::math::NOT\_A\_NUMBER

const double NOT\_A\_NUMBER

(Quiet) not-a-number value.

**Definition:** constants.hpp:53

stan::agrad::var::val

double val() const

Return the value of this variable.

**Definition:** var.hpp:209

stan::agrad::var::vi\_

vari \* vi\_

Pointer to the implementation of this variable.

**Definition:** var.hpp:40

stan::agrad::vari

The variable implementation base class.

**Definition:** vari.hpp:28

var.hpp

stan::agrad::precomp\_v\_vari

**Definition:** precomp\_v\_vari.hpp:11

stan::agrad::var

Independent (input) and dependent (output) variables for gradients.

**Definition:** var.hpp:27

constants.hpp

operator\_unary\_negative.hpp


---

[ Stan Home Page ]

*© 2011–2014,
Stan Development Team.*
